# Choice of 16S ribosomal RNA primers affects the microbiome analysis in chicken ceca

**DOI:** 10.1038/s41598-021-91387-w

**Published:** 2021-06-04

**Authors:** Nadia Darwish, Jonathan Shao, Lori L. Schreier, Monika Proszkowiec-Weglarz

**Affiliations:** 1 Agricultural Research Service, NEA, Animal Biosciences and Biotechnology Laboratory, United States Department of Agriculture, 10300 Baltimore Avenue, B-200, Rm. 100B, BARC-East, Beltsville, MD 20705 USA; 2 Agricultural Research Service, Northeast Area, Statistic Group, United States Department of Agriculture, Beltsville, MD 20705 USA

**Keywords:** Microbiology, Molecular biology

## Abstract

We evaluated the effect of applying different sets of 16S rRNA primers on bacterial composition, diversity, and predicted function in chicken ceca. Cecal contents from Ross 708 birds at 1, 3, and 5 weeks of age were collected for DNA isolation. Eight different primer pairs targeting different variable regions of the 16S rRNA gene were employed. DNA sequences were analyzed using open-source platform QIIME2 and the Greengenes database. PICRUSt2 was used to determine the predicted function of bacterial communities. Changes in bacterial relative abundance due to 16S primers were determined by GLMs. The average PCR amplicon size ranged from 315 bp (V3) to 769 bp (V4–V6). Alpha- and beta-diversity, taxonomic composition, and predicted functions were significantly affected by the primer choice. Beta diversity analysis based on Unweighted UniFrac distance matrix showed separation of microbiota with four different clusters of bacterial communities. Based on the alpha- and beta-diversity and taxonomic composition, variable regions V1–V3(1) and (2), and V3–V4 and V3–V5 were in most consensus. Our data strongly suggest that selection of particular sets of the 16S rRNA primers can impact microbiota analysis and interpretation of results in chicken as was shown previously for humans and other animal species.

## Introduction

Bacteria are the major component of chicken gastrointestinal tract (GIT) microbiota that plays important role in health, nutrition, host physiology regulation, GIT development, and growth. Microbiota composition and function can be affected by age, host genotype and sex, diet composition and form, dietary ingredients such as probiotics, prebiotics, synbiotics, phytobiotics and bacteriophages, stress, antibiotics, and GIT location^1–3^. Recently, the number of available data characterizing the avian microbiota has significantly increased^4^. Published papers mostly focused on the impact of diet^5,6^, disease^7,8^, antibiotics^5^, probiotics^7,9,10^, prebiotics^11,12^ and environmental exposures^13^ on the chicken microbiota. Analysis of the microbiota is believed to be important to improve animal nutrition strategies, animal health, and well-being. In chickens, a diverse microbiota is found throughout the GIT with the most diversity in the cecum which serves as a key organ for fermentation of various forms of polysaccharides to short-chain fatty acids^14,15^.

Historically, microbiota in GIT was detected by biochemical, microbiological, immunological, and molecular biology techniques^16^. Because most of the microbiota in GIT is strictly anaerobic, it was difficult to identify and characterize individual species using classic methodology^16^. With time, more sophisticated molecular biology methods were developed to characterize microbiota, including PCR, denaturing gradient gel electrophoresis (DGGE), temperature gradient electrophoresis (TGGE), microarrays, and next-generation sequencing (NGS)^17,18^. Recently, the microbial community profiling method based on the 16S ribosomal RNA (rRNA) sequencing approach (NGS) has become the most popular to determine the taxonomic composition and diversity of chicken microbiota^19^. Bacterial 16S rRNA contains 9 hypervariable regions used to calculate evolutionary relationships and similarities between species, that are flanked by highly conserved regions which are generally used to design polymerase chain reaction (PCR) primers^20^. The 16S rRNA profiling consists of many steps such as: sample collections and storage, DNA isolation, 16S primer selections, 16S rRNA PCRs, libraries preparations and indexing, sequencing, raw data analysis (pipeline or software selection), OTU/ASV (Operational Taxonomic Unit/Amplicon Sequence Variant) picking, database selection, diversity analysis, and statistical analysis. Several bioinformatics pipelines for raw sequences analysis has been developed and used to provide a taxonomic composition and population diversity including Mothur^21,22^ and QIIME^23–25^. In the case of taxonomic composition, most analyses are performed using databases such as Greengenes^26^, the Ribosomal Database Project^27^, and SILVA^28^.

Even though 16S profiling is the most popular approach to study microbial diversity, it is characterized by several limitations including amplicon size, primer sensitivity, amplification errors, and contamination^29^. It has been already shown that primer design^30–32^, library preparation^33^, DNA isolation methods^34,35^, and PCR amplification artifacts can introduce unique biases that can affect community structure, richness, and microbial population analysis^36^ and lead to over- or under-representation of individual bacteria within communities^37^. Moreover, different sequencing platforms and bioinformatics pipelines can affect the average relative abundance of microbiota and shape the taxonomic community profiles^31,38^. Additionally, the Microbiome Quality Control project (MBQC) in human microbiome study reported that the DNA isolation method, as well as 16S rRNA primers used, are the major sources of variation, with sequencing depth and sample storage having a smaller but detectable influence on the data^39^.

In chicken microbiota studies, even though the experiments are commonly standardized and based on identical breeds, the results are often contradictory and the results depend on used animal (breed, age, gender, etc.), the experimental design (feeding and sampling), and DNA extraction and sequencing methods. Therefore, it is hard to compare those data and correlate them with each other^19,40^. The development of a standardized protocol for microbiota profiling in chickens, similar to the one used in human microbiota research, has been proposed by Borda-Molina and colleagues^40^ to obtain comparable data sets for poultry microbiota.

Following the above recommendation and taking into account the fact that microbial studies in poultry, covered, so far, the V1–V3, V3–V4, V4–V5, V4–V6, V1, V3 or V4 region of the 16S rRNA gene^3,41–45^, the present study aimed to explore the influence of applying the different sets of 16S rRNA primers on chicken microbiota diversity, taxonomic composition and predicted function.

## Results

### Sequencing

A total of 12 samples obtained from chicken cecal content at three different ages (n = 4 for age) were used in 16S rRNA high-throughput sequencing using eight different 16S rRNA primer sets. Chicken DNA amplification with these primer sets resulted in averaged indexed PCR product size ranging from 315 bp (V3) to 769 bp (V4–V6) (data not shown). In all cases, a single PCR band was visible on the electropherogram, but the intensity of the PCR band was different among primers sets, with the lowest one for V3 and the highest one for V1–V3(1), V3–V4 and V4–V5, and V3–V5 (data not shown). Sequencing of 12 samples generated 16,050,150 sequences with 15,776 to 939,976 sequences per sample. After removing chimeric sequences, the total pool of sequences was reduced to 11,113,440 reads with 13,524 to 634,783 sequences per sample.

### Microbiota diversity analysis

**Figure 1:**
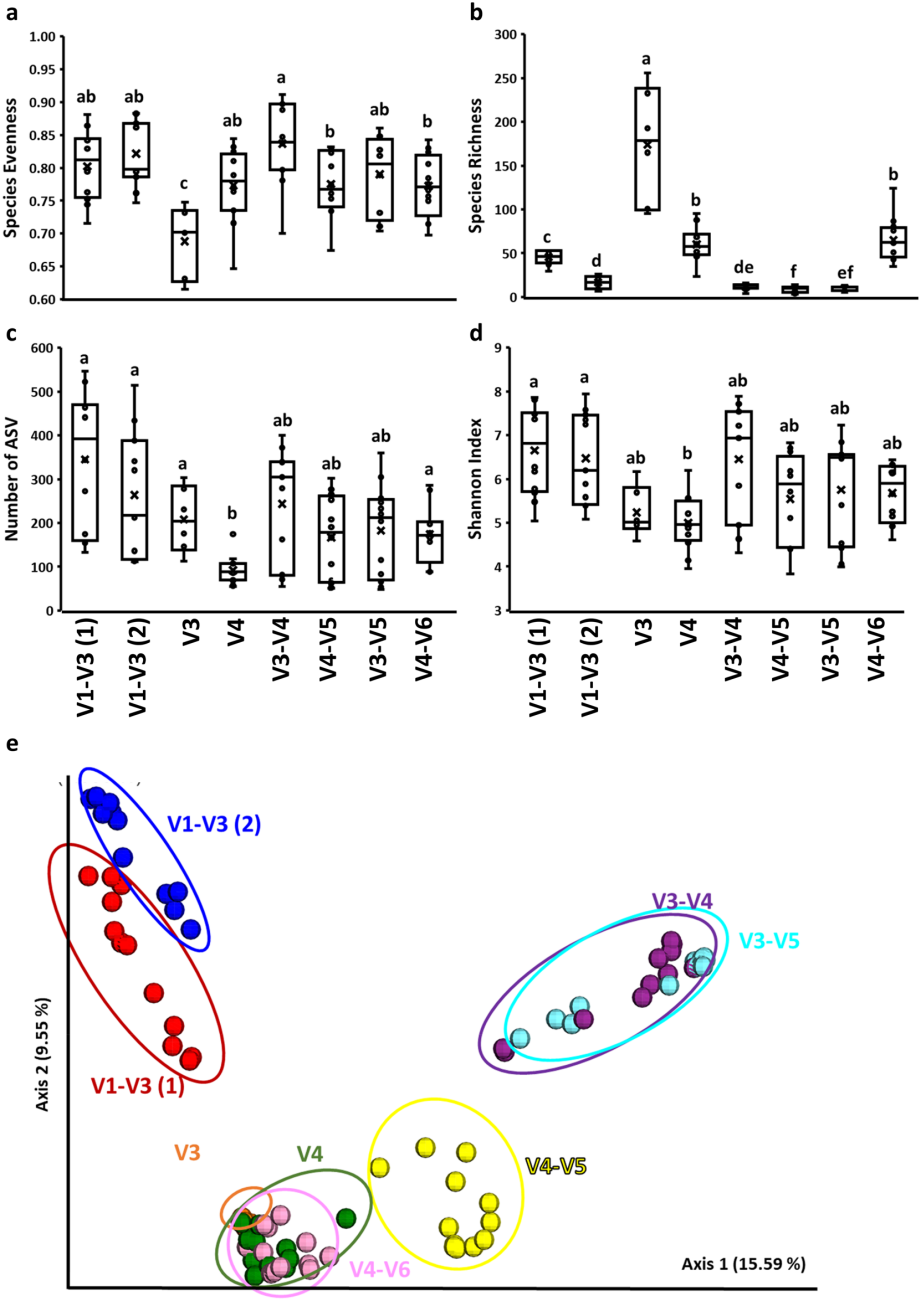
Effect of primer set on alpha **(a–d)** and beta **(e)** diversities in cecal microbiota. Alpha diversities indices: **(a)** Evenness, **(b)** Richness, **(c)** ASV number, and **(d)** Shannon index. **(e)** Principal coordinate analysis (PCoA) based on unweighted pairwise UniFrac distances between primer sets in cecal microbiota. ^a,b,c,d,e,f^q < 0.05 (q represents *p* value corrected for false discovery rate).

Significant (*P* < 0.05) differences in alpha diversity indices in chicken cecal microbiota were observed when different 16S rRNA primers were used (Fig. 1). In the case of Evenness, the V3 set was characterized by the lowest one in comparison to other primer sets while V3–V4 showed the highest Evenness in comparison to V4–V5 and V4–V6 (Fig. 1a). In contrast, V3 was characterized by the highest Richness while V4–V5 by the lowest Richness (Fig. 1b). The number of ASVs was significantly (*P* < 0.05) lower in V4 in comparison to V1–V3, V3, and V4–V6 sets (Fig. 1c). Finally, the Shannon index, which represents community Richness and Evenness, was the least affected by primer set showing only significant differences between V1-V3 and V4 primer sets (Fig. 1d). Beta diversity analysis (Principal Coordinate Analysis, PCoA) based on Unweighted UniFrac distances as well as PERMANOVA analysis revealed clear clustering of microbial communities due to primer choice (Fig. 1e). Five different clusters were formed, with V1–V3(1), V1–V3(2), V3–V5 and V3–V4, V4–V5, and V3, V4, and V4–V6 separated from each other.

### Taxonomic composition

**Figure 2:**
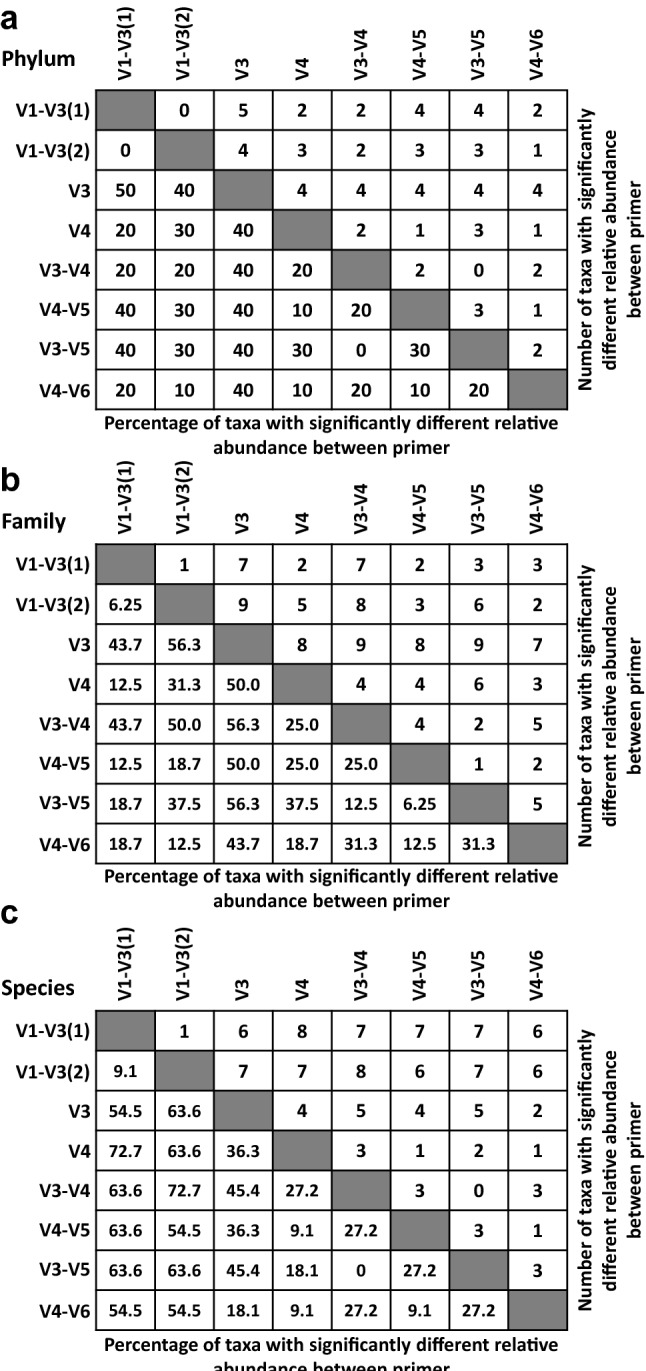
Number and percentage of taxa significantly different in relative abundance level between primer pairs. **(a)** phylum level, **(b)** family level and **(c)** species level. Number of taxa that were characterized by significant differences in relative abundance were counted between primer pairs. The percentage of the taxa were calculated for each primer pair comparison: (number of taxa that were significantly (*P* < 0.05) different in relative abundance level/total number of taxa for each taxonomical level) × 100.

**Figure 3:**
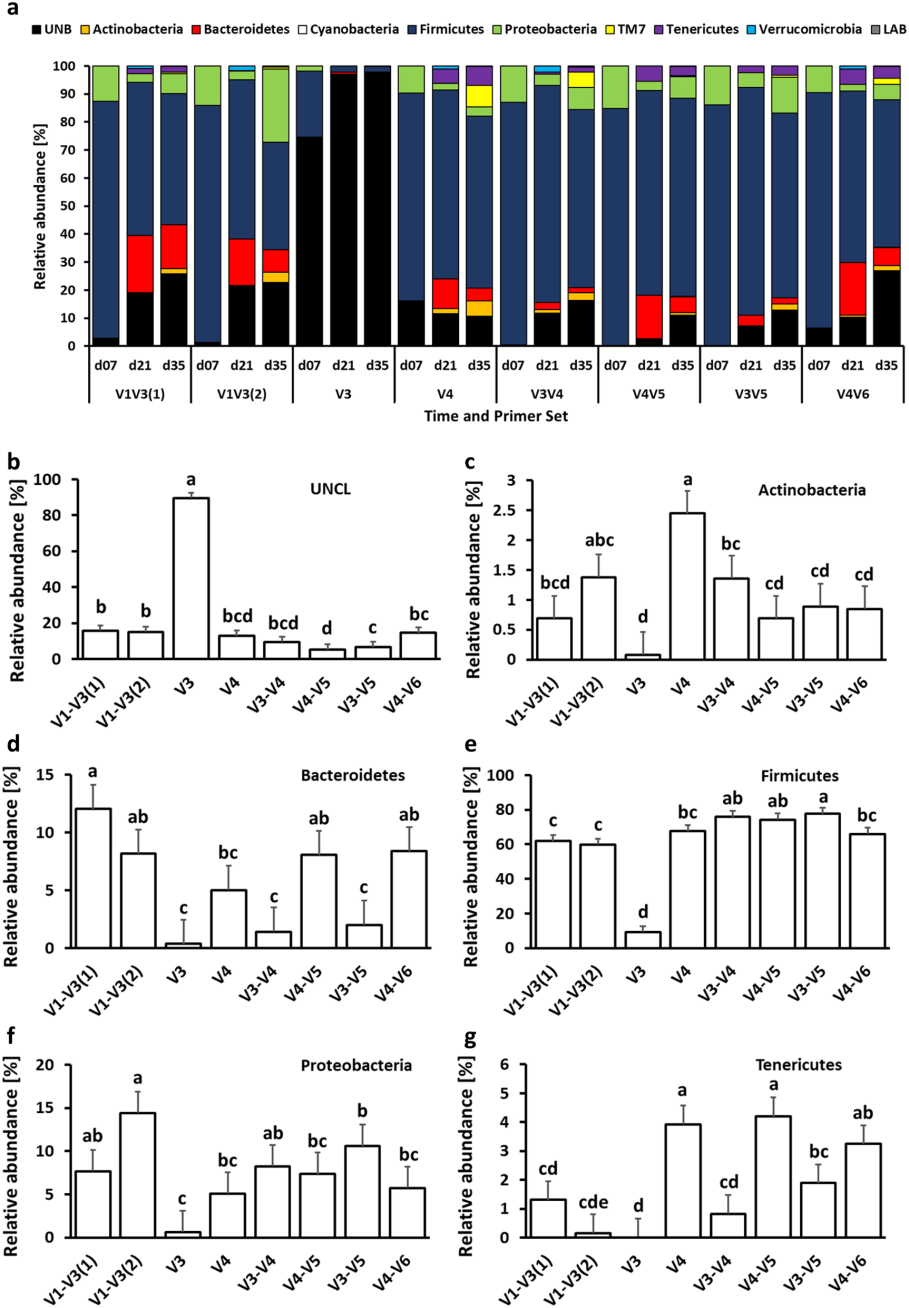
Changes in relative bacterial abundance (%) in chicken cecal content at phylum level. **(a)** Taxonomic profile of chicken cecal microbiota. Effect of primer sets on the relative abundance of **(b)** unclassified bacteria (UNCL), **(c)** Actinobacteria, **(d)** Bacteroidetes, **(e)** Firmicutes, **(f)** Proteobacteria, and **(g)** Tenericutes. Only significant (*P* < 0.05) data are shown and only first 6 phyla with the highest abundance are shown. Different letters denote statistically significant (*P* < 0.05) differences between primer sets.

**Figure 4:**
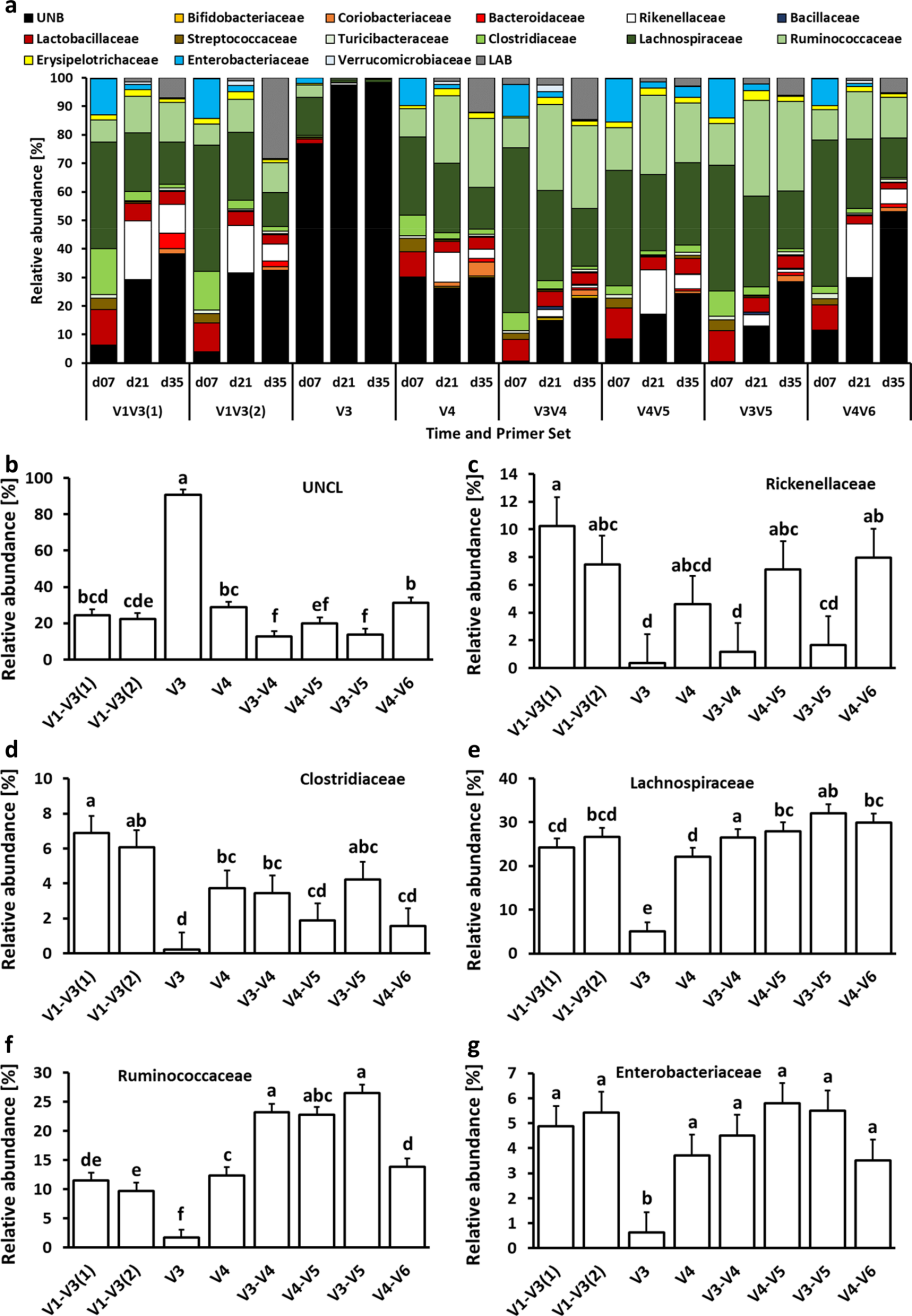
Changes in relative bacterial abundance (%) in chicken cecal content at family level. **(a)** Taxonomic profile of chicken cecal microbiota. Effect of primer sets on the relative abundance of **(b)** unclassified bacteria (UNCL), **(c)** *Rikenellaceae*, **(d)** *Clostridiaceae*, **(e)** *Lachnospiraceae*, **(f)** *Ruminococcaceae*, and **(g)** *Enterobacteriaceae*. Only significant (*P* < 0.05) data are shown and only first 6 families with the highest abundance are shown. Different letters denote statistically significant (*P* < 0.05) differences between primer sets.

**Figure 5:**
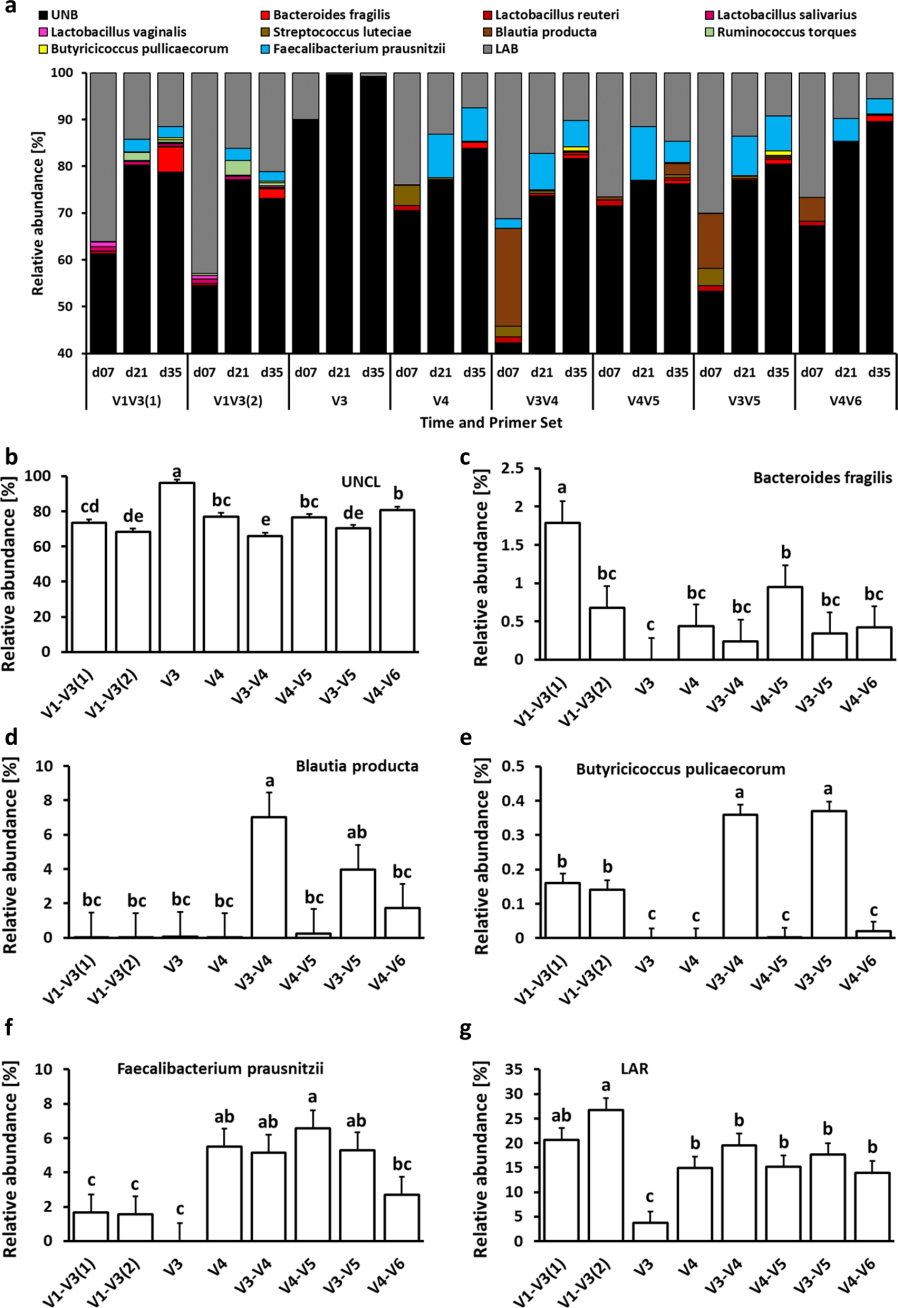
Changes in relative bacterial abundance (%) in chicken cecal content at species level. **(a)** Taxonomic profile of chicken cecal microbiota. Effect of primer sets on the relative abundance of **(b)** unclassified bacteria (UNCL), **(c)** *Bacteroides fragilis*, **(d)** *Blautia producta*, **(e)** *Butyricicoccus pulicaecorum,* **(f)** *Faecallibacterium prausnitzii*, and **(g)** low abundance reads (LAR). Only significant (*P* < 0.05) data are shown and only first 6 species with the highest abundance are shown. Different letters denote statistically significant (*P *< 0.05) differences between primer sets.

Figure 2 depicts the number and the percentage of detected features for phylum (Fig. 2a), families (Fig. 2b), and species (Fig. 2c) that were significantly different in relative abundance between primer pairs. On the phylum level, no differences were observed between V1–V3(1) and V1–V3(2) primer pairs while V3 primers were characterized by the greatest number of different taxa in comparison to the V1–V3(1) primer pair (Fig. 2a). At the family level, the lowest differences in sets were observed for V1–V3(1) vs. V1–V3(2) and V4–V5 vs. V3–V5 primer pairs, while the biggest differences in relative abundance were observed for V1–V3(2) vs. V3, V3 vs. V3–V4, and V3 vs. V3–V5 primer pair comparison (Fig. 2b). Finally, at the species level, no significant differences in relative abundance were observed for V3–V4 and V3–V5 primer pair comparison. The biggest differences in the relative abundance of species were observed between V1–V3(1) and V4, and V1–V3(2) and V3–V4 primer sets (Fig. 2c). Changes in taxonomic composition on phylum, family, and species level due to primer set are presented in Figs. 3, 4 and 5. At the phylum level, chicken cecal microbiota was composed predominantly (except for the V3 primer set) of Firmicutes, Proteobacteria, and Bacteroidetes with a lower relative abundance of Actinobacteria, TM7, Tenericutes, and Verrucomicrobia (Fig. 3a). Besides changes in microbial composition due to the age of birds, the selection of 16S primers has a significant effect on microbiota composition in chicken ceca. The first six phyla with the highest relative abundance that were significantly (*P* < 0.05) affected by the primer set are presented in Figs. 3b–g. Primer set V3 was characterized by the highest abundance of unclassified bacteria and the lowest abundance of other phyla. The highest relative abundance of Actinobacteria was obtained with V4 primers (Fig. 3c) while Bacteroidetes relative abundance was the lowest in the case of V3, V3–V4, and V3–V5 primer sets (Fig. 3d). The Firmicutes relative abundance ranged from 60% (V1–V3) to 75% (V3–V4, V4–V5, and V3–V5) (Fig. 3e). The highest Proteobacteria level was detected with V1–V3(2) primer set (Fig. 3f) while the highest Tenericutes level was detected with V4 and V4–V5 primer sets (Fig. 3g). A similar pattern was observed at the family level (Fig. 4). V3 primer set was characterized by the highest (P < 0.05) abundance of Unclassified bacteria (UNCL) and the lowest relative abundance of other bacterial families in chicken ceca (Fig. 4b). The highest abundance families that show significant differences due to primer set selection are presented in Fig. 4c–g. The selection of V3, V3–V4, and V3–V5 led to the lowest abundance level of the *Rickenellaceae* family in chicken ceca (Fig. 4c). The highest *Clostridiaceae* level was presented with V1–V3(1) primers (Fig. 4d) while a relatively stable level of *Lachnospiraceae* family was observed regardless of primers except for V3 and V4 primer set (Fig. 4e). The highest relative abundance of the *Ruminococcaceae* family was detected with V3–V4, V4–V5, and V3–V5 primer sets (Fig. 4f). No changes in abundance of *Enterobacteriaceae* were detected among 16S primers except for V3 primers (Fig. 4g). Regardless of the primer set, the taxonomic composition at the species level was characterized by a high abundance level of UNCL (Fig. 5a) ranging from 60% to almost 90% (Fig. 5b). Changes in the abundance level of bacterial species in chicken ceca are presented in Fig. 5c–g. Similarly to the phylum and family level, taxonomic composition on species level was affected by primer sets. The highest abundance level of *Bacteroides fragillis* was only detected with a V1–V3(1) primer set (Fig. 5c) while the high abundance level of *Blautia producta* was detected only with V3–V4 (Fig. 5d). In the case of *Butyricocccus pullicaecorum*, the highest abundance level was observed for V3–V4 and V3–V5 primer sets followed by V1–V3 sets (Fig. 5e). Primer sets of V4, V3–V4, V4–V5, and V3–V5 led to the detection of the highest abundance of *Faecallibacterium prausnitzii* (Fig. 5g). Also, the level of low abundance reads (LAR) was significantly affected by primer set choice (Fig. 5g).

### Microbiota predicted function

**Figure 6:**
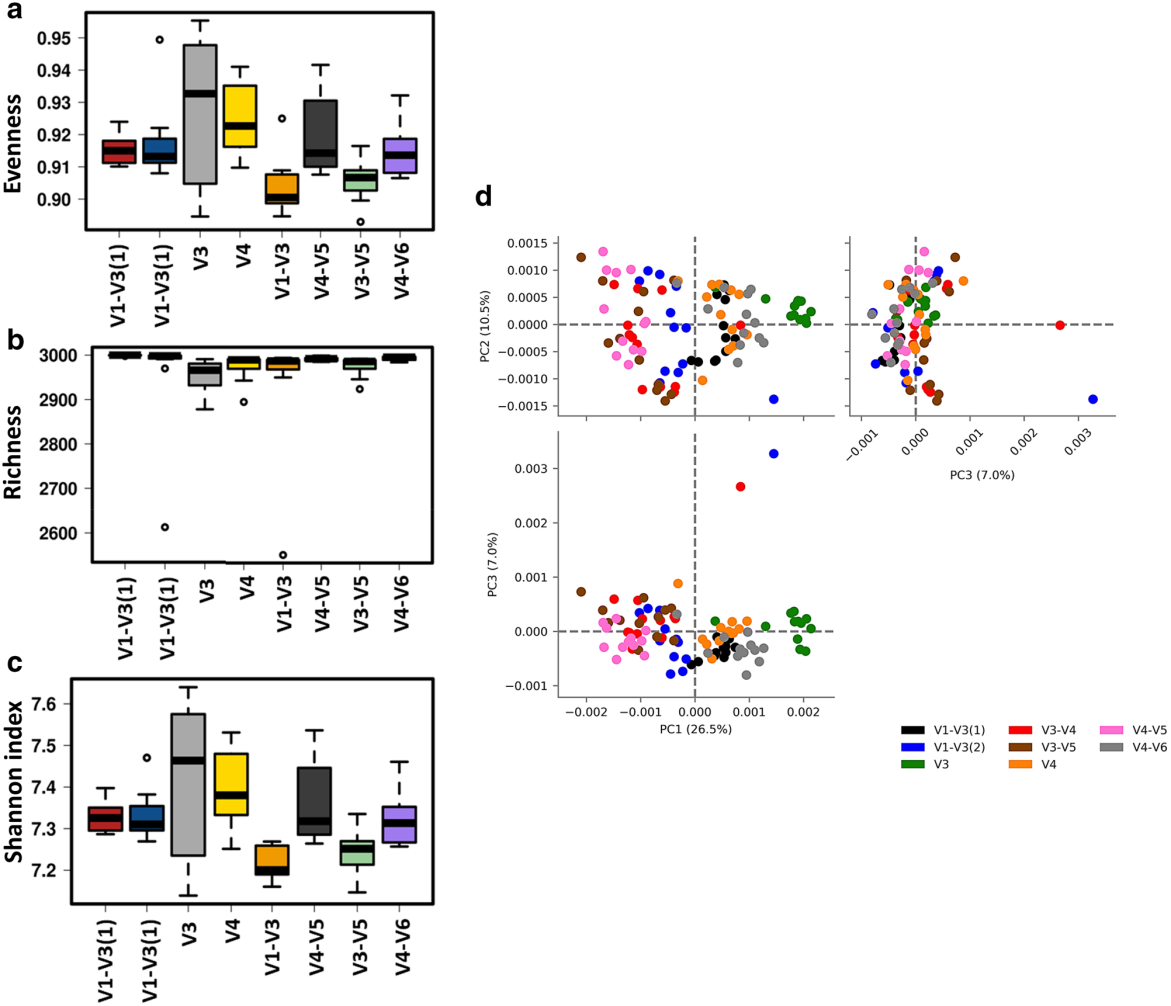
Effect of the primer set on alpha and beta diversities of predicted function of the cecal microbiota in chickens. Function of the microbiota was determined using PICRUST and visualized using Calypso **(a–c)** and STAMP **(d)**. Alpha diversities: **(a)** evenness (*P* < 0.001), **(b)** richness (*P* = 0.33) and **(c)** Shannon index (*P* < 0.0001) **(d)** beta diversity between primer set bacterial populations was determined using principal component analysis (PCA).

**Figure 7:**
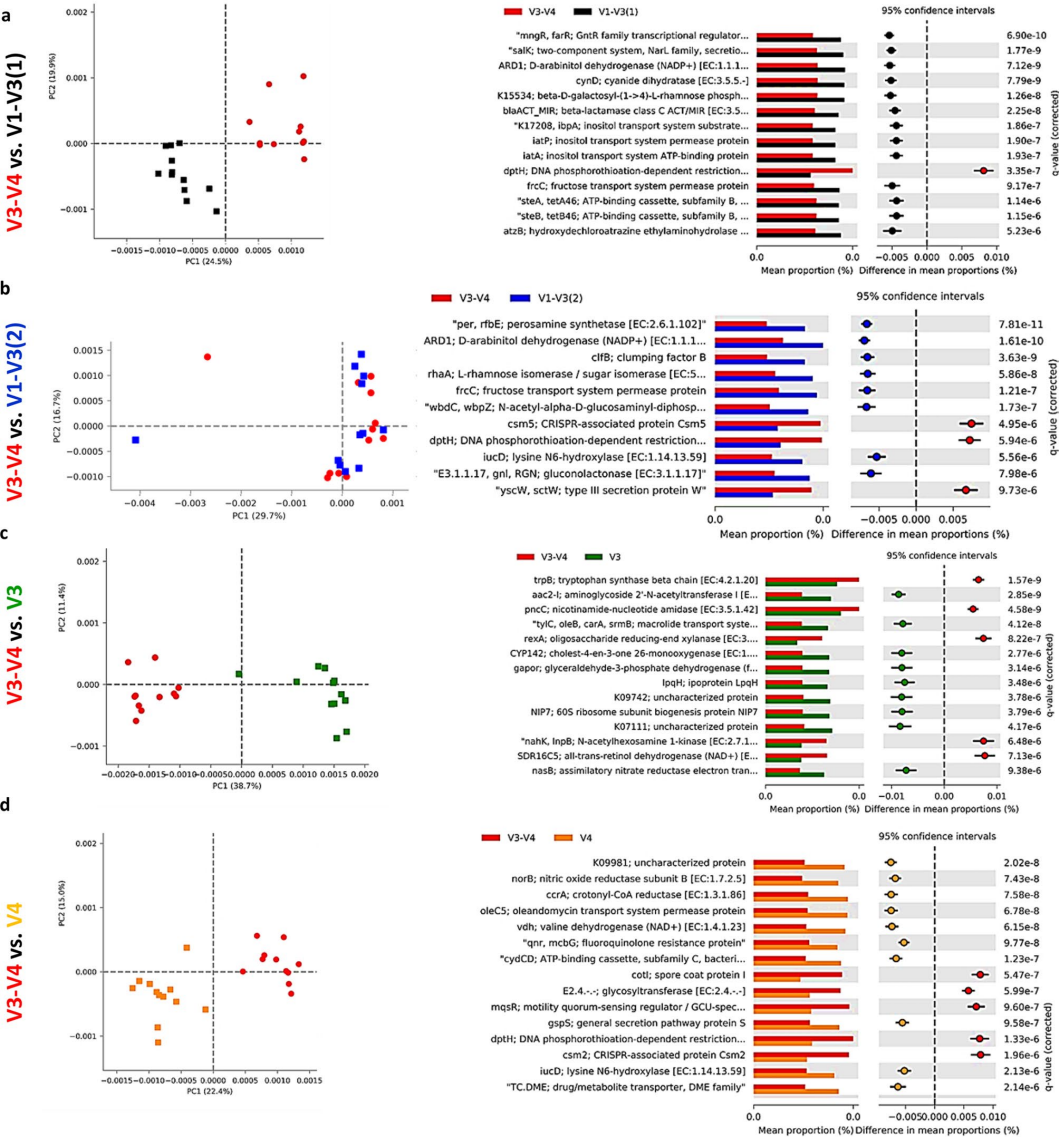

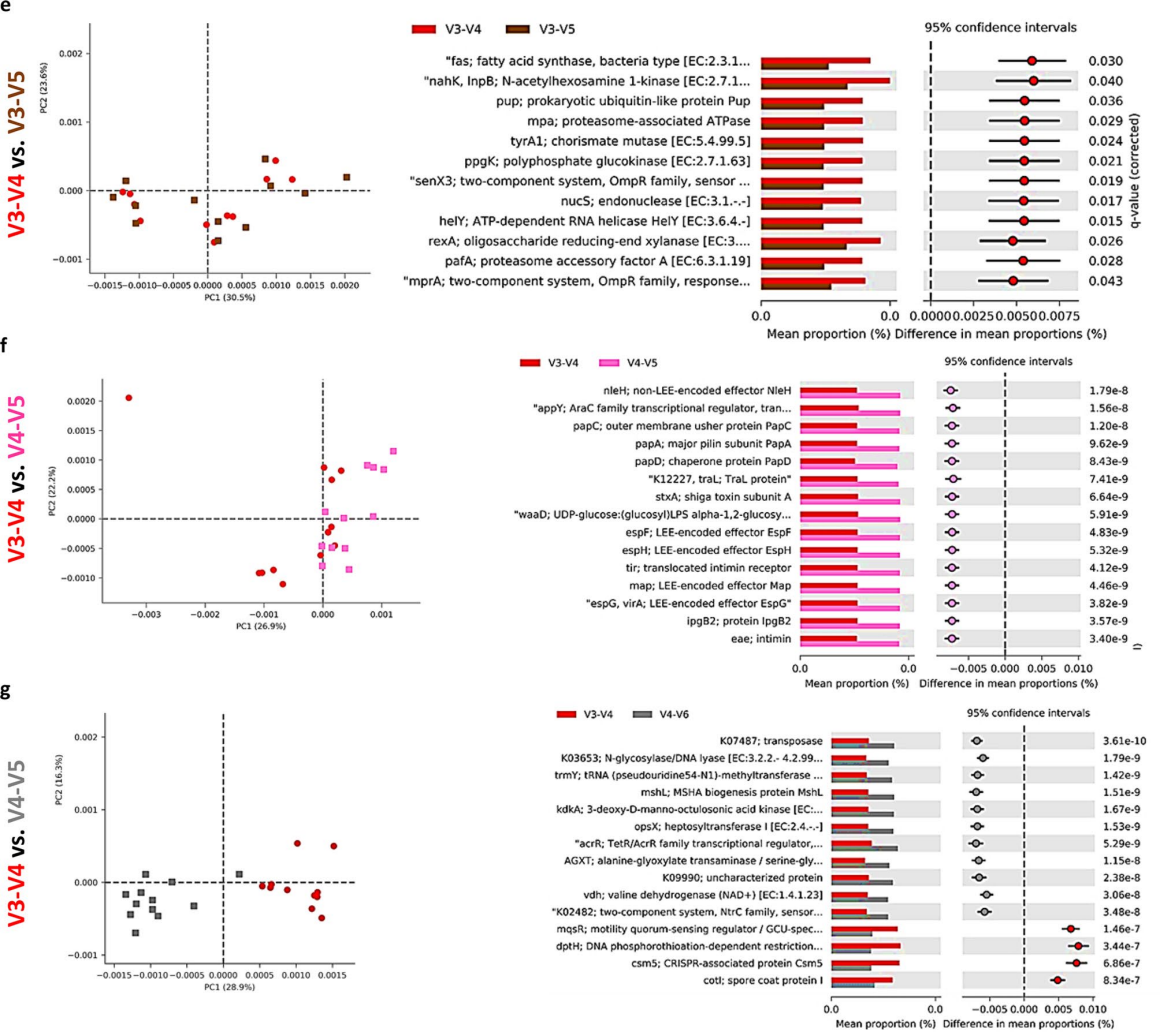
Effect of the primer set on predicted function of the cecal microbiota in chickens. Function of the microbiota was determined using PICRUST with KEGG database and visualized using STAMP. Principal component analysis (PCA) and extended error bar plots for comparison of predicted function of microbiota between **(a)** V3–V4 and V1–V3(1) **(b)** V3–V4 and V1–V3(2), **(c)** V3–V4 and V3, **(d)** V3–V4 and V4, **(e)** V3–V4 and V4–V5, **(f)** V3–V4 and V3–V5, and **(g)** V3–V4 and V4–V6 primer sets.

Alterations in the presumptive function of the cecal microbiota in broiler chicken due to different 16S primers were evaluated using PICRUSt2 analysis and visualized using Calypso and STAMP. Evenness and Shannon index of predicted function (Fig. 6a–c) was significantly (*P* < 0.001) affected by the primer set while Richness was not affected (*P* = 0.33). Similarly, Principal Component Analysis (PCA) showed separation between bacterial function due to the primer set (Fig. 6d). Figure 7 shows PCA analysis and the changes in the top predicted microbial genes between V3-V4 and other primer sets. We choose to compare all other sets to the V3–V4 primer set because it is the most widely used primer set and almost exclusively used by our laboratory to determine the taxonomic composition of chicken microbiota. Comparison of V3–V4 with V1–V3(1) primer sets, revealed clear function separation (PCA) with most of the top function upregulated with V1–V3(1) primer set (Fig. 7a). In contrast, no separation of predicted function was detected for V3–V4 and V1–V3(2) sets, but most of the top function were upregulated with V1–V3(2) primer set (Fig. 7b). Both comparisons, V3–V4 vs. V3 and vs. V4 showed a similar pattern of changes with clear separations between these two populations as shown by PCA and upregulations of 2/3 of top KEGG function in V3 and V4 primer sets (Fig. 7c,d). V3–V4 and V3–V5 function comparison revealed no clustering (PCA) with all the top function being upregulated in the V3–V4 primer set (Fig. 7e). Similarly, V3–V4 and V4–V5 samples were clustered together but all top of KEGG function were upregulated in the V4–V5 primer set (Fig. 7f). Definite clustering of function between V3–V4 and V4–V6 primers were detected and the top of KEGG functions were upregulated in V4–V6 primers in comparison to V3–V4 primers (Fig. 7g).

## Discussion

Over the last decade, several regions of the 16S gene were used to characterize the chicken microbial population, including V1–V2^46,47^, V1–V3*^3,48^, V3^3,49^, V4^3,7,50^, V3–V5^51,52^, V4–V6^3^, V4–V5^53^, V3–V4^5,54^, and V1–V9^55^. At the same time, many studies on mammalian, human, or environmental samples raised the issues of data bias introduced by methodology, including primer choices for 16S^30–32^. Moreover, the call for standardization of microbiota profiling protocol has been issued in the human microbiome community^39^, but recently also among poultry microbiome researchers^40,56^. The MBQC project that analyzed the effects of experimental sample collection, nucleic acid extraction, sequencing protocol, and bioinformatic approaches on 16S profiling of the human fecal microbiota, determined that the extraction method, as well as 16S rRNA primers selection,  are the main sources of data fluctuation^39^. There is an evident need for a similar project in the poultry microbiome community since every step in the microbiome sequencing protocol such as sample collection, DNA isolation, and library preparation, can introduce bias to the data. Little attention so far has been drawn on the possible data bias due to methodology issues or bioinformatic analysis for poultry microbiome analysis.

To gain a deeper understanding of how the 16S primer choice can influence the final results for microbiota diversity and composition, we tested 8 different 16S primer pairs located in 7 different regions of the 16S rRNA in broiler chicken cecal content. Two primer sets were located in the V1–V3 region and they differed only by few base pairs, and one pair was located within V3 and V4 region. The remaining 4 pairs span the V3–V4, V4–V5, V3–V5, and V4–V6 regions of the 16S rRNA. Alpha and beta diversities analysis clearly showed huge differences between these primer sets. It is interesting, that only a few base pair differences in a primer set designed for the same 16S region (V1–V3(1) vs. V1–V3(2)) resulted also in a significant difference for Richness index and they clustered separately when PCoA was employed. Our data are in agreement with previously published studies. Zhao et al.^3^, using microbiome differences between high and low body weight chickens, described the difference in the number of species detected among V3, V4, V1–V3, and V4–V6 primer pairs. Moreover, V3 has been shown to underestimated species Richness^3^, while the V4 primer provides estimates similar to these obtained by the full length of 16S^20^ and V4 primers showed the greatest similarity to community profiling determined by shotgun sequencing^30^. In contrast to our studies, Bhogoyu and colleagues^55^ have examined different regions (V1–V9) in chickens and determined that most of the reads (60%) were located in very conserved V3 region followed by V7–V8, V7, V8 (~ 14%), V4 (8.7%) and V9 and V2 region (from 1.2 to 2.2%). Other studies have shown that primers can target certain strains allowing for non-proportional amplification of specific populations^57,58^. Targeting V3–V4 regions favor enteric pathogens and gut microorganisms^56^. Moreover, in silico study by Kim et al.^59^ identified V1–V3 and V1–V4 regions to be targeted for analysis of bacteria.

Hugerth and Andersson^58^ have shown that the choice of primers is very sensitive since the same bacterial community amplified with different high-quality primer pairs can give a different microbiological profile. Indeed in our studies, we have shown that primer choice had a significant effect on taxonomic composition in chicken ceca. On the phylum level, Actinobacteria were favored by the V4 primer set while Bacteroidetes were detected at a very low level with V3, V3–V4 and V3–V5 primer pairs. Similar significant changes were detected at the family and species level. On the species level, *Butyricicoccus pulliceacorum* was detected at a high abundance level only by V3–V4 and V3–V5 primer pairs while *Blautia producta* was favored by V3–V4 primers. It has been also shown that taxonomic composition (classification) relies more on primer choice than the sequencing platform^30^. Additionally, we have shown that the predicted function of the microbiota differs among 16S primer sets. These data clearly indicate that the primer set has a significant role in the determination of taxonomic composition and that the employment of different primers may lead to a different interpretation of results. The same conclusion was stated by others suggesting that the selection of specific hypervariable regions for 16S primers will influence microbiome data and subsequent interpretation^60,61^. Moreover, studies of others showed that data generated by different 16S primers are not directly comparable^62–64^. There are no universal primers, so always bias will be introduced by selecting primer pairs^30^, therefore it would be perfect if we had a standardized protocol for microbiome studies in poultry and could apply the same primer set across all studies to be able to compare data sets between studies and discuss the data with published literature. Moreover, Trembley and colleagues^30^ suggest that protocol consistency, particularly the primer choice, is more important in comparative 16S studies than the specific primers. On the other hand, Allali et al.^38^ confirmed differences between sequencing platforms and libraries preparation protocols in the determination of microbial diversity and species richness in their studies but they also suggested that the same biological conclusions could be drawn from data as long as the data are collected and analyzed consistently throughout the course of the experiment.

In conclusion, similarly to previous reports of primer bias in human and other animal datasets, we have clearly shown similar data bias due to the 16S primer choice in chicken samples. Moreover, be believe, that our results might help researchers make an informed decision about which variable region should be selected for analysis of chicken microbiota samples. This is only one factor that can affect the data generation and interpretation. Our data also showed a need for a standardized protocol for 16S studies in poultry. If the protocol is not developed, one of the possible solutions for this problem would be to study parallel regions of 16S rRNA^65^, but this may not be the most cost-effective solution in poultry research.

## Methods

### Animals, experimental protocols, and tissue sampling

All animal care procedures were approved by the USDA-ARS Institutional Animal Care and Use Committee. The study was carried out in compliance with ARRIVE guidelines (https://arriveguidlines.org/arrive-guidelines). All methods were carried out in accordance with relevant guidelines and regulations. Ross 708 broiler chicken (Longenecker’s Hatchery, Elizabethtown, PA) were raised from hatch to day 35 in-floor pen settings. One hundred and fifty-two hatchlings were equally distributed between 4-floor pens, covered with wood shavings, and equipped with heat lamps, nipple drinkers, and feeders. All birds had full access to a commercial type corn-soybean meal-based diet (starter from day 1 to day 21 or grower (day 21 to day 35) that met or exceeded all NRC^66^ recommendations as well as average nutrient usage concentrations in the US for 2012^67^. Cecal content samples were collected from birds (one per pen) 7, 21, and 35 days post-hatch to determine the luminal bacterial population. Isolated specimens were snap-frozen in liquid nitrogen and stored at − 80℃ until bacterial DNA isolation.

### DNA isolation and library preparation

DNA was extracted from cecal contents and evaluated as described previously^68^. The 16S rRNA amplicon libraries were generated according to Illumina’s workflow and chemistry (Illumina, Inc., San Diego, CA). Eight PCR primer sets targeting different regions of the variable region of the 16S rRNA (Table 1) were used. All selected primer sets were previously published and used in poultry research. Amplicon PCR followed by index PCR, and PCR amplicon cleaning were performed as described previously^68^. Concentration and quality of the amplicons were determined using QIAxcel DNA Hi-Resolution cartridge, proprietary QIAxcel ScreenGel software (version 1.6.0, https://www.qiagen.com), and QIAxcel Advanced System (Qiagen) per manufacturing instructions. Library pooling, dilution, denaturation, and sequencing were performed as described in Proszkowiec-Weglarz et al.^68^. The 16S rRNA gene sequences determined in this study were deposited in the NCBI Sequence Read Archive (SRA) database (SRA accession # PRJNA680391).

**Table 1:** 16S rRNA primers used to analyze microbiota diversity, composition and function in chicken cecal content.

Name	Region	Forward primer (5’ → 3’)	Reverse primer (5’ → 3’)	References
V1–V3(1)	V1–V3	DAGAGTTTGATCMTGGCTCAG	TMTTACCGCGGCNGCTGGCAC	Daquigan et al., 2016^69^
V1–V3(2)	V1–V3	GAGAGTTTGATYMTGGCTCAG	ACCGCGGCTGCTGGCAC	Li et al., 2018^48^
V3	V3	GATCCTACGGGAGGCAGCA	CTTACCGCGGCTGCTGGC	Cao et al., 2018^49^
V4	V4	GTGCCAGCMGCCGCGGTAA	GGACTACHVGGGTWTCTA T	Zhang et al., 2018, Xu et al., 2018^7,50^
V3–V4	V3–V4	CCTACGGGNGGCWGCAG	GACTACHVGGGTATCTAATCC	Klindworth et al., 2013^77^
V4–V5	V4–V5	GTGCCAGCMGCCGCGGTAA	CCGTCAATTCMTTTRAGTTT	Wang et al., 2018^53^
V3–V5	V3–V5	CCTACGGGAGGCAGCAG	CCGTCAATTCMTTTRAGT	Lucke et al., 2013^78^
V4–V6	V4–V6	GTGCCAGCMGCNGCGG3	GGGTTNCGNTCGTTG	Zhao et al., 2013^3^

### 16S rRNA data processing and analysis

The quality of the raw reads was determined using the FASTQC software (https://www.bioinformatics.babraham.ac.uk/projects/fastqc/). Raw sequences were trimmed of adapters and low-quality reads and cleaned using the BBDuk program as part of the BBTools software suite (BBMap—Bushnell B.—sourceforge.net/projects/bbmap/), using the following parameters: with parameters ktrim=r k=23 mink=11 hdist=1 tpe tbo qtrim=rl trimq=10. BBMerge (part of the BBTools suite) was used to determine if sequences in the pair-end library merged using the default settings. An optimization table was created for paired-end and single-end reads for each primer set to determine the best parameters of non-chimeric data. Based on the optimization table, only single-end (forward) sequences were imported into Quantitative Insight Into Microbial Ecology (QIIME) software package 2 (version 2018.12.0, http://qiime2.org)^24^ to perform quality control and analysis of the sequence reads. Demultiplexed, single-end sequence data were denoised with DADA2. Naïve Bayesian classifier was used for taxonomic classification against the Greengenes database v13_8 (http://greengenes.secongenome.com). A sampling depth of 12,480 was used for alpha and beta diversity analysis. QIIME data were transformed using R package Compositions^72^ followed by Phylogenetic Investigation of Communities by Reconstruction of Unobserved States (PICRUSt)2^73^ was used to predict metagenome pathways for each primer set using the Kyoto Encyclopedia of Genes and Genomes (KEGG)^74^. Statistical Analysis of Metagenomic Profiles (STAMP)^75^ and Calypso software^76^ (cgenome.net, version 8.84) was used to create a visualization of metabolic pathway comparison.

### Statistical analyses

Microbiome composition data were obtained by normalization to the total number of reads in each sample (relative abundance) and were analyzed using two-way (time and primer set) ANOVA using GLM (SAS, ver. 9.4). Significance was set at *P* < 0.05. Differences between alpha diversity indices were tested using the Kruskal–Wallis test (QIIME2). The difference in community structure due to main effects (time and primer set) and their interaction were statistically tested by non-parametric multivariate ANOVA (PERMANOVA) with 999 permutations using QIIME 2 software package. Within STAMP analysis, ANOVA followed by post-hoc Turkey-Kramer test^77^, corrected for False Discovery Rate (FDR, Benjamini–Hochberg analysis^78^) was used for multiple group comparison while two group comparison was performed using Welsh t-test^77^ with Benjamini-Hochber FDR analysis^78^.

## Data Availability

The 16S rRNA gene sequences determined in this study were deposited in the NCBI Sequence Read Archive (SRA) database (SRA accession #PRJNA680391).
